# Oxygen Defects Containing TiN Films for the Hydrogen Evolution Reaction: A Robust Thin-Film Electrocatalyst with Outstanding Performance

**DOI:** 10.3390/nano14090770

**Published:** 2024-04-27

**Authors:** Ayoub Laghrissi, Mohammed Es-Souni

**Affiliations:** 1 Currently at Mads Clausen Institute, University of Southern Denmark, 6400 Sønderborg, Denmark; laghrissi@mci.sdu.dk; 2 Institute of Materials and Surface Technology, Honorary Member of Kiel University of Applied Sciences, 24249 Kiel, Germany

**Keywords:** hydrogen evolution reaction, titanium nitride, oxygen defects, TiNO, linear sweep voltammertry, DFT

## Abstract

Density functional theory (DFT) calculations of hydrogen adsorption on titanium nitride had previously shown that hydrogen may adsorb on both titanium and nitrogen sites with a moderate adsorption energy. Further, the diffusion barrier was also found to be low. These findings may qualify TiN, a versatile multifunctional material with electronic conductivity, as an electrode material for the hydrogen evolution reaction (HER). This was the main impetus of this study, which aims to experimentally and theoretically investigate the electrocatalytic properties of TiN layers that were processed on a Ti substrate using reactive ion sputtering. The properties are discussed, focusing on the role of oxygen defects introduced during the sputtering process on the HER. Based on DFT calculations, it is shown that these oxygen defects alter the electronic environment of the Ti atoms, which entails a low hydrogen adsorption energy in the range of −0.1 eV; this leads to HER performances that match those of Pt-NPs in acidic media. When a few nanometer-thick layers of Pd-NPs are sputtered on top of the TiN layer, the performance is drastically reduced. This is interpreted in terms of oxygen defects being scavenged by the Pd-NPs near the surface, which is thought to reduce the hydrogen adsorption sites.

## 1. Introduction

The share of renewables in the energy mix has seen a steep increase in the last decade, primarily driven by environmental concerns. In many regions of the world, the share of renewables is now at least 20% and is expected to increase during the coming decade to 40 or even 50% [1,2]. Recent developments in Li-ion battery modules that became much more competitive, as Li price on the world market continues to fall, have made it possible to efficiently store energy and release it to the grid in case of fluctuations in solar and wind power [3]; this certainly has contributed to the widespread acceptability of renewables by commercial stakeholders. Nevertheless, an efficient exploitation of renewables will also have to consider other energy storage possibilities, such as (super)capacitors and water electrolysis [4,5], to mention only the electrochemical methods. In particular, water electrolysis, mainly for the production of green hydrogen via the hydrogen evolution reaction (HER), is highly attractive and could afford a powerful way for energy storage that is clean and sustainable and could be used even in remote and desertic regions.

HER is a well-known electrochemical interfacial process that essentially depends on pH, catalyst, and applied voltage. The reaction mechanisms and kinetics involved at the interface between electrolyte and catalyst have been described in detail in numerous review articles, for example, articles by Conway and Tilak [6], Bockris and Conway [7], and Eley et al. [8], to mention only a few. The generally accepted reaction steps using a metal (*M*) catalyst under acidic conditions are [6,7,8] as follows:

(1) $$H_{3}O^{+}+M+1e^{-}\to MH_{ads}+H_{2}O\;\;(\mathrm{Volmer step})$$

followed by reactions (2) and/or (3)

(2) $$H_{3}O^{+}+MH_{ads}+1e^{-}\to M+H_{2↑}+H_{2}O\;\;(\mathrm{Heyrovsky step})$$

(3) $$2MH_{ads}\to H_{2↑}+2M\;\;(\mathrm{Tafel step})$$

Practically, the Tafel Equation (4) is used to evaluate the catalyst’s performance as follows:

(4) $$U_{0}=\beta log\frac{J}{J_{0}}$$

where *U*_0_ is the hydrogen overpotential, *β* and *j*_0_ are temperature-dependent material constants, and j is the measured current. *β* = 2.3 RT/αF, where R is the gas constant, T is the absolute temperature, F is the Faraday constant, and α is a constant. *j*_0_ is the exchange current density, representing the equilibrium exchange current density of the forward and reverse reactions at the electrode. From Equation (4), we may infer that the larger *j*_0_ and α, the smaller the overpotential *U*_0_, a measure of a catalyst’s performance.

The mechanisms of HER have been rationalized by different authors in terms of the rate-determining mechanisms (Equations (1)–(3)), considering the different acting kinetic parameters such as adsorbed hydrogen surface coverage, overpotential, and pH (e.g., Conway and Tilak [4], Bhardwaj et al. [9], Shingawa et al. [10]). A critical parameter that has been largely discussed in the literature is the hydrogen adsorption energy, E_C-H_, on the catalyst’s (C) surface, which governs the hydrogen surface coverage and the rate-limiting step of diffusion. Together with the exchange current density, *j*_0_, from the Tafel equation, E_C-H_ has been used to evaluate catalysts via the so-called volcano plot [10,11,12,13]. In simple terms, one may state that a moderate E_C-H_ and a higher *j*_0_ may constitute meaningful material selection criteria for catalyst development. To date, Pt has an unchallenged position at the top of the volcano, precisely because of the criteria mentioned above, and any new electrode material will be measured against the performance of Pt. However, Pt is a precious, strategic noble metal with limited resources, a fact that has triggered a spate of research for alternative materials. Several catalysts have been reported in the literature for the HER, including transition metals and various compounds, as compiled in the review article by Eftikhari [11]. Catalyst performance is generally weighed in terms of the magnitude of overpotential necessary for HER at a given pH, the current density generated/weight of the active catalyst, and durability/stability. As mentioned above, Pt, which is mostly used in acidic conditions, is well known for its outstanding performance [14,15,16,17]. In particular, Pt-NPs are the best known HER catalysts to date, performing at very low overpotentials. Noble metal-free catalysts such as Ni and Ni-Mo [18], metal compounds, including FeS_2_, MoS_2_ [19], and metalloid compounds such as C_3_N_4_ [20], to mention only a few of them, have been reported with acceptable performances, although stability issues have arisen. Also, while some of the catalysts mentioned above may perform well in tiny batches, their scalability is questionable as multiple processing steps are involved, which complicates quality management and the reproducibility of the results. New and efficient noble-metal-free HER catalysts that can be scaled up using well-established industrial processes are therefore of critical importance, as they will cut electrode costs.

In the present study, the potential application of TiN as an HER catalyst is considered. TiN is a well-established multifunctional material with applications spanning hard-coating for steel tools to plasmonics [21,22,23]; it is usually processed on an industrial scale as a thin film of various thicknesses using physical (PVD) or chemical (CVD) vapor deposition (e.g., review articles [24,25]). Further, it has been shown using density functional theory that hydrogen adsorbs on TiN, with the adsorption energy being dependent on termination (N or Ti) and adsorption sites [26,27]. The values obtained on TiN (111) range from 0.60 to 3.11 eV for N-termination and from 1.93 to 3.59 eV for Ti termination [26]. A slightly different value was obtained by Siodmiak et al. on the TiN (100) surface [27]. Overall, the adsorption energies are considered moderate, albeit larger than on Pt. The diffusion barrier along the crystal sites was also shown to be rather low. This would unveil promising non-noble metal electrocatalyst electrodes that are chemically and mechanically resistant. More advantages lie in the well-documented fact that they can be processed on an industrial scale using versatile and commercially available industrial equipment. It will be shown in this paper that TiN thin films of approximately 400 nm, deposited on Ti substrates via reactive gas sputtering, are potent HER catalysts in acidic media with current densities in the range of −70 mA/cm^2^ at an overpotential of −0.1 V vs. SHE, which is in the range of the performance of Pt/C. This performance is primarily ascribed to the existence of oxygen defects on the surface. It will also be indirectly shown that the TiN surface adsorption sites are critical for HER; a few nanometers of sputtered Pd lead indeed to a drastic decrease in performance.

## 2. Materials and Methods

### 2.1. Synthesis and Characterization

Commercially pure, grade 1 titanium sheets, 0.1 mm thick, were purchased in an annealed, oxide-scale-free, and straightened condition from Goodfellow (Goodfellow, Hamburg, Germany). They were cut into 5 × 5 cm^2^ samples, degreased in 99.9% ethanol in an ultrasonic bath, rinsed twice with ethanol, and dried with compressed air before introduction into the PVD chamber (PVD75, Lesker, Jefferson Hills, PA, USA; electron beam system from Ferrotec GmbH, Unterensingen, Germany). An adhesion layer of 2 nm Ti was magnetron sputtered on the substrate surface using a Ti-target. On top of the heterostructure above, the TiN layers were processed via reactive ion sputtering using 99.995 pure titanium pellets (Lesker) and high-purity nitrogen (99.9999%, Westfalen AG, Münster, Germany). The following conditions were found to yield nearly stoichiometric TiN: electron beam evaporation of Ti at 8.75 kV and 65 to 90 mA in a mixture of nitrogen and argon at a ratio of 8/15 and a pressure of 2 to 5 × 10^−3^ Torr. Under these conditions, the sputtering rate was in the range of 0.18 to 0.21 nm/s. The total sputtered thickness was 400 nm. The layer thickness was cross-checked on cross-sections of coated Si that were introduced together with the Ti substrates.

The microstructure and morphology of the samples were characterized with a high-resolution scanning electron microscope (SEM Ultra Plus, ZEISS, Oberkochen, Germany) operating in the secondary (SE) and energy-selective backscattered (ESB) electron modes. The SEM is also equipped with an energy-dispersive X-ray spectroscopy (EDS) package (INCAx-act, Oxford Instruments, High Wycombe, UK). For the purpose of achieving better quality SEM micrographs, a thin Au film, approximately 2–3 nanometers in thickness, was sputtered onto the samples. The structure was characterized by X-ray diffraction (XRD, X’Pert Pro diffractometer, PANalytical, Eindhoven, The Netherlands) in grazing incidence diffraction mode with constant θ = 1° using monochromatic CuKα radiation with λ = 1.5418 Å. The device has a full-width to half-maximum resolution of 0.03°.

An electrochemical workstation (ZAHNER IM6e, Kronach, Germany) was used for linear sweep voltammetry (LSV) measurements from −0.8 V to 0.3 V. The electrochemical experiments were performed at a sweep rate of 50 mV/s in a 0.5 M H_2_SO_4_ solution at a pH of 0.36, using a three-electrode set-up with a Pt mesh and HydroFlex (a reversible H_2_ reference electrode) as counter and reference electrodes, respectively. The ratio of the working electrode (WE) area to that of the counter electrode (CE) was approximately 1 to 4. All potentials were referenced to the reversible hydrogen electrode (RHE). The current was normalized by the sample’s measured area. All the H_2_SO_4_ solutions were saturated with the forming gas N_2_ + H_2_ (after the usual bubbling with nitrogen) before measurement.

### 2.2. DFT Calculations

**Figure 1:**
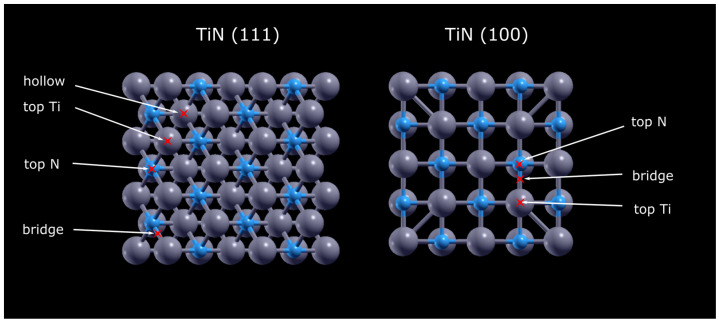
Top view of modeled TiN (100) and TiN (111) with possible adsorption sites.

Using the Quantum ESPRESSO v.6.0 package [28,29,30], ab initio calculations based on density functional theory were conducted to calculate charge distribution and hydrogen adsorption on 111- and 100-oriented TiN surfaces. In the present study, electro-ion interactions were described using projector augmented wave (PAW) potentials, while exchange correlations were represented using generalized gradient approximations (GGA) based on Perdew–Burke–Ernzerhof (PBE) frameworks [31]. A 400 eV plane wave basis cut-off energy was set for the initial optimization of the structures by relaxing atomic positions using the Broyden–Fletcher–Goldfarb–Shanno (BFGS) method. To achieve high accuracy, a self-convergence field convergence criterion of 10^−6^ electrons and 10^−5^ for the total force was used. The core correction and occupation were smearing and Gaussian. A k-point grid size of 5 × 5 × 5 was utilized for self-consistent field and lattice optimization calculations, while a denser grid of 10 × 10 × 10 was employed for non-self-consistent calculations of the density of states. In Figure 1, hydrogen adsorption is shown in various locations, namely, top N, top Ti, bridge for TiN (100), and topTi, topN, bridge, and hollow for TiN (111). Oxygen defects were introduced by replacing nitrogen atoms with oxygen, following the formula Ti_(1−x/3)_Ti_(vac)x_N_1−x_O_x_, where x is set to 0.1. This specific concentration of oxygen ensures a calculated change in crystal structure that mainly affects the surface layer, while the bulk layers remain unchanged [32]. This selective modification aims to explore changes in surface properties, such as reactivity and electronic structure, without significantly altering the overall properties of the material. The introduction of oxygen to the surface layer of TiN (111) represents a focused approach to modifying material properties for targeted applications. To achieve the most stable surface hydrogen distance, the hydrogen atom and the top layer of the plate were allowed to relax their atomic positions. To calculate the hydrogen adsorption energies, the usual definition of Equation (5) is used as follows:

(5) Eads=EMetal−H−(Emetal+12EH2)

## 3. Results

### 3.1. Morphology Structure and Chemistry

**Figure 2:**
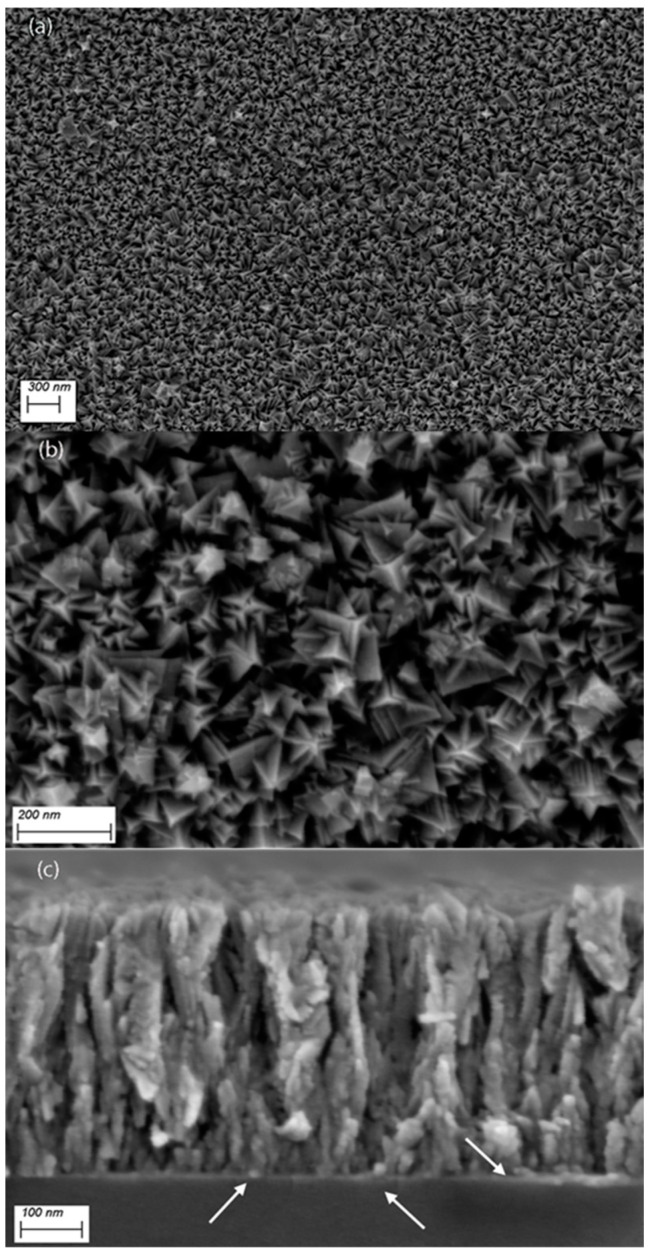
Top-view secondary electron (SE) micrographs of the TiN layers on Ti-substrate (**a**,**b**) show, at two different magnifications, the particular morphology of the poly-nanocrystals with their faceted and rugged appearance. (**c**) A cross-section micrograph obtained on a silicon substrate that was coated under the same conditions. Notice the perpendicular growth from high-density nucleation sites at the interface (arrows).

**Figure 3:**
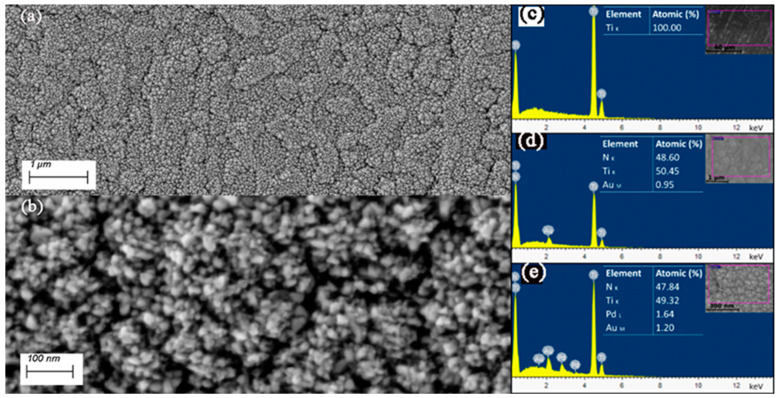
SE micrographs of the Ti-TiN-Pd layer at low (**a**) and high magnification (**b**) show Pd-NPs topping the TiN crystal spikes. The right images show EDS analysis of Ti (**c**), TiN (**d**), and Ti-TiN-Pd (**e**).

Large-area, homogeneous TiN films are obtained on Ti-sheets using the experimental conditions depicted in the experimental section. A photograph of a 10 × 10 cm^2^ sample is shown in Figure S1 (Supplementary Materials). The morphology of the TiN layers is displayed in Figure 2. The top-view SEM micrographs in Figure 2a,b show faceted star-like-shaped nanocrystals that seem to build up individually, independent from one another, and leading to a highly porous, rugged surface. This morphology may arise from the different growth velocities of differently oriented nanocrystals. Note that the pyramidal crystals (predominantly in 111 orientation) top the surface (apparent from their brighter contrast in the secondary electron micrographs, which arises from their higher proximity to the in-lens detector). The cross-sectional micrograph of Figure 2c indeed shows that the poly-nanocrystals grow perpendicular to the interface from highly dense nucleation sites, conferring to them a columnar morphology. One might also see that the crystals are in many areas separated by voids. As shown in the XPS results, oxygen defects are present in the TiN layer, probably incorporated during reactive sputtering owing to residual oxygen pressure in the chamber. As divalent oxygen ions replace trivalent nitrogen ions, Ti^3+^ vacancies are created to ensure electric neutrality (see below for discussion). The presence of these defects entails specific properties of Ti(N,O)_x_ that are different from those of TiN or TiO_2_, although the rock salt structure remains roughly unaltered [32]. To qualitatively assess the impact of the surface defects on HER, a thin Pd layer of approximately 10 nm was sputtered on top of the TiN layer in a second experiment, as shown in Figure 3. This should completely or partially saturate the Ti(N,O)_x_ surface sites with Pd, a known HER catalyst.

**Figure 4:**
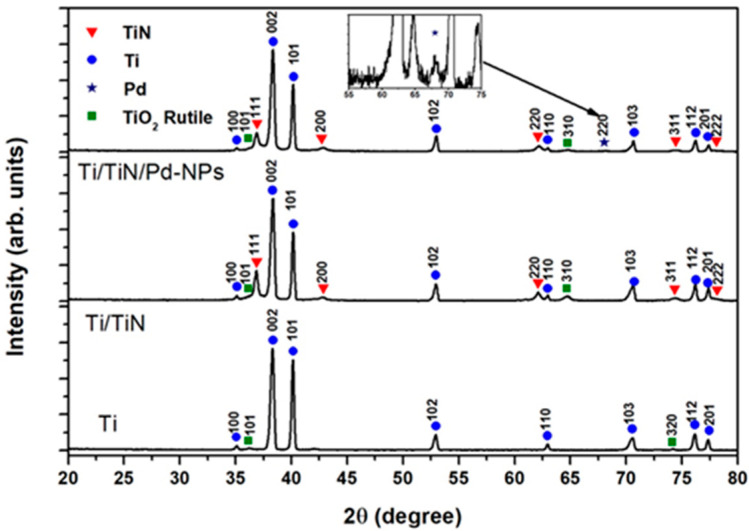
XRD patterns of the substrate Ti, Ti/TiN, and the Ti/TiN-PdNPs composite layer. The TiN pattern is indexed according to the PDF card # 01-087-0630 for the non-stoichiometric phase TiN_0.88_ (see below for discussion). The inset in the upper pattern is an enlargement of the pattern containing the 220 Pd reflex (*).

The XRD patterns are displayed in Figure 4. There are traces of the TiO_2_-rutile phase on/in the Ti sheet, which might originate from earlier processing. The reflexes pertaining to TiN could be assigned to the non-stoichiometric TiN_0.88_ phase (PDF card no.: 01-087-0630), with the 111 orientation being predominant. Also, in this sample, traces of rutile might be present but are difficult to ascertain as the peaks are faint and the most intense rutile reflexes (R110) could not be detected. The sample with Pd-NPs on top of the TiN layer shows only one faint Pd reflex, but EDS analysis is conclusive about the presence of Pd, as shown in Figure 3e.

**Figure 5:**
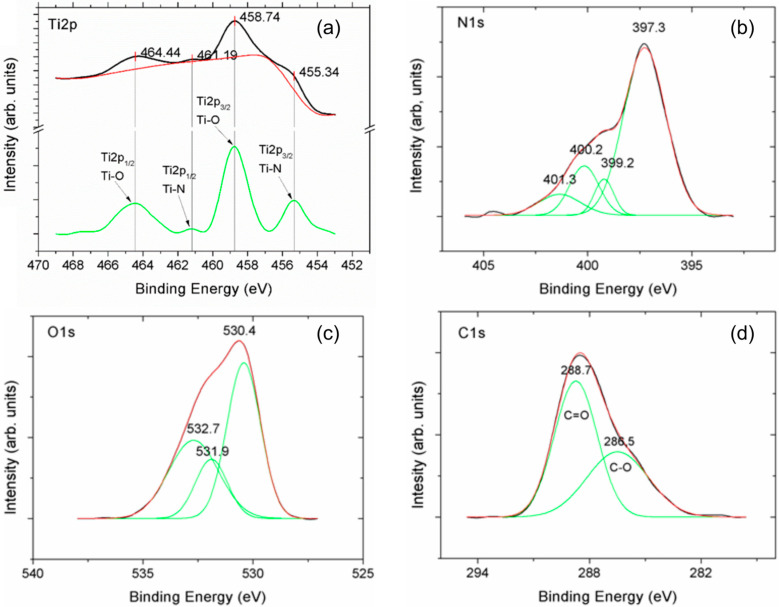
XPS of Ti/TiN showing the core levels of Ti2p, N1s, O1s, and C1s (see main text for more details). The measured Ti2p in (**a**) is deconvoluted in the green curve, showing the bonding energies of the different compounds. N1s (**b**), O1s (**c**), and C1s (**d**) are discussed above (see also Table 1).

To investigate the surface chemistry of the TiN layer, X-ray photoelectron spectroscopy measurements were performed after argon ion cleaning. The high-resolution XPS peaks of Ti2p, N1s, O1s, and C1s are displayed in Figure 5. The carbon 1s peak arises from surface contamination and cannot be associated with any compound at the surface. The doublets Ti2p_1/2_ and Ti2p_3/2_ of the Ti2p peak show energy levels that can be attributed to Ti-O in TiO_2_ and Ti-N in TiN bonds. The deconvoluted high-resolution XPS curves show peak positions that are in large agreement with those reported by others [32,33,34,35]. Ti2p shows energy levels that can be unambiguously assigned to Ti-O in TiO_2_ and Ti-N in TiN bonds. The N1s and O1s peaks may be more informative about the different species present at the surface. Table 1 shows the binding energies for those peaks derived from Figure 5 and the corresponding assignments. There is no doubt as to the presence of oxides at the surface of the TiN layer; we suggest these oxides to be most probably in the form of TiO_2_ and defective TiO_y_N_x_. Notwithstanding this fact, oxygen defects are probably present in the TiN_x_ bulk as well, not least because, in the processing method used, oxygen cannot be wholly eliminated from the chamber, leaving sufficient oxygen partial pressure for the formation of the defects observed.

### 3.2. HER Investigations

**Figure 6:**
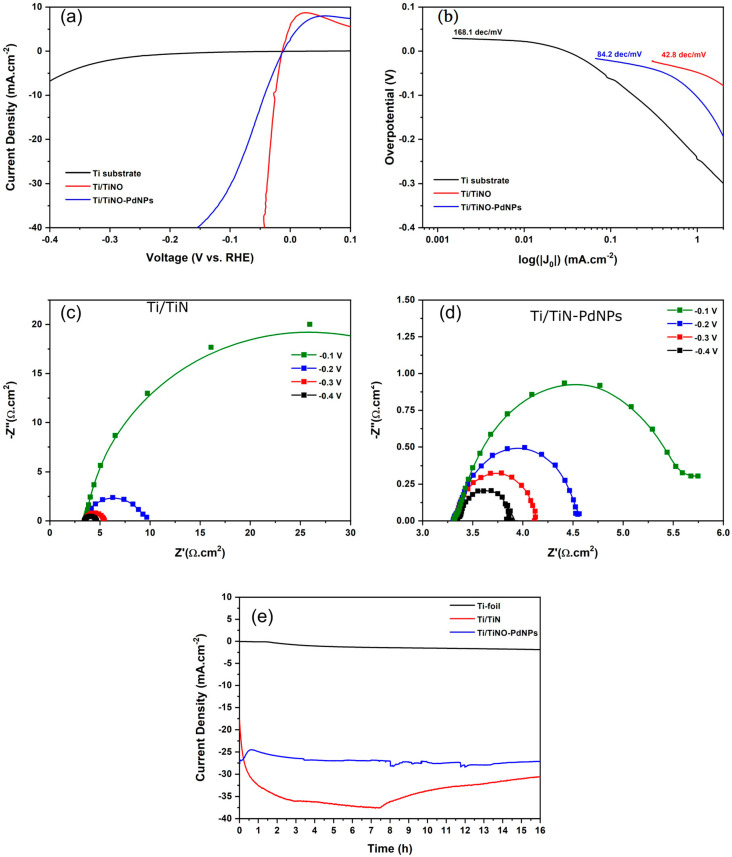
(**a**) IR-corrected linear sweep voltammograms in 0.5 M H_2_SO_4_ of TiNO and TiNO-PdNPs. (**b**) Tafel plots corresponding to the LSV curves. The current density is normalized by the geometric surface area. Electrochemical impedance spectroscopy at different overpotentials of −0.1, −0.2, −0.3, and −0.4 V for (**c**) TiNO and (**d**) TiNO-PdNPs. (**e**) Non-corrected chronoamperometric curves in 0.5 M H_2_SO_4_ at −0.4 V for 16 h.

The linear sweep voltammetry (LSV) curves are shown in Figure 6a for the plain Ti-substrate, Ti/TiNO, and Ti/TiNO-PdNPs. There is undeniably a huge difference in the HER behavior, depending on the sample chemistry. While the Ti-substrate rather shows a flat LSV curve with faint activity (Ti is known for its activity for the HER at high overpotentials) [36], a drastic increase in the current density is observed with the TiNO layer, which shows almost two orders of magnitude higher values (in comparison to Ti, e.g., at −0.1 V). The overpotential for the HER is also drastically reduced and rivals the values reported for the best known C/Pt-NPs catalysts [37]. This activity is also higher than what is reported in the literature for TiN nanowires (NW) in 1 M HClO_4_ [38]. When a thin Pd layer is sputtered on TiNO, there is a conspicuous decrease in the current density and an increase in the overpotential in comparison to pristine TiNO (e.g., approximately −2 mA/cm^2^ in comparison to −40 mA/cm^2^ for Ti-TiNO at an overpotential of −0.05 V), as shown in Figure 6a. Considering the EIS spectra in Figure 6c,d, it is readily seen that for both electrodes, the data are well described by an almost perfect half-circle, suggesting a reactive system consisting of a series resistance, R_s_ (the ohmic resistance of the electrolyte), and a charge transfer resistance, R_ct_, in parallel with a capacitance element [39].

The data unambiguously show that the overall resistance, R_s_ + R_ct_, of the Ti/TiN-PdNPs electrode is substantially lower than that of pristine TiNO, regardless of the applied overpotential, as presented in Table 2. It may then be stated that the lower performance of the Ti/TiNO-PdNPs electrode cannot be imputed on an increase in R_ct_ and that other interfacial effects, probably related to the electronic structure of the surface, might be responsible (see below for discussion).

The Tafel plots are shown in Figure 6b, with the indicated slopes corresponding to the low overpotential region. The slope of TiNO is in the vicinity of the values usually observed for Pt-NPs, which points to a Tafel mechanism (Equation (3)), controlled by the Volmer step (Equation (1)) [4,5,6]. The almost double slope characterizing the Ti/TiNO-PdNPs rather hints to a Heyrovsky-like mechanism, with the HER being primarily dependent on the rate of hydrogen desorption (Equation (2)).

The chronoamperometric measurements over a period of 16 h are displayed comparatively in Figure 6c. The current density of the Ti/TiNO electrode first increases (in absolute value) during the first 7 h of polarization, followed by a steady decrease with final saturation at a value of 30.5 mA/cm^2^, which is slightly higher than the value at the start of the measurement. In the case of the Ti/TiNO-PdNPs electrode, the current density first decreases in the first hour before stabilizing at a value of 27 mA/cm^2^, which is roughly the value at the beginning of the measurement.

After the HER measurements, microscopic characterizations of the microstructures were performed; there were no noticeable changes to any of the samples investigated. This is well documented in Figure S2 (Supplementary Materials), where both morphology and chemical analyses are displayed.

## 4. Discussion

### 4.1. HER of Oxygen Defects Containing TiN

The properties depicted above will now be discussed briefly. The surface chemistry of the TiN layer undoubtedly plays a key role in tuning the properties of this material. There is consensus that oxygen spontaneously, that is, exothermally, substitutes for nitrogen, giving rise to oxygen defects, O_N_, when its partial pressure exceeds 10^−12^ Torr. In the case of oxygen-rich atmospheres, the possibility of interstitial oxygen defect (O_i_) formation has also been put forward using DFT [40]. In our case, where the base pressure before sputtering was 2 × 10^−8^ Torr, the defects that formed were probably of the O_N_ type. Because divalent oxygen is substituting trivalent N in the TiN lattice, electrical neutrality is established via the formation of Ti^3+^ vacancies, Ti_V_^3+^. The general stoichiometry can be expressed as Ti_(1−x/3)_^3+^Ti_(v)x/3_^3+^N_1−x_^3−^O_x_^2−^, where x may take values from 0 to 1 [32]. The impact of oxygen defects on the functional properties of TiN has been well documented and ranges from effects on its plasmonic and electronic properties to band-gap shift, depending on oxygen content [32,40]. Concerning the topic dealt with presently, there has been no report detailing the effects of oxygen defects on the HER activity of TiN, which stresses the novelty of the present results. Oxygen defects are thought to affect the HER properties by altering the hydrogen adsorption/desorption properties and the concentration of adsorption sites, although a detailed investigation of the oxygen defect concentration on HER is still to be conducted. As mentioned earlier, the introduction of oxygen defects in the TiN lattice entails the formation of Ti_(1−x/3)_^3+^Ti_(v)x/3_^3+^N_1−x_^3−^O_x_^2−^, which alters the electronic properties of the surrounding atoms.

**Figure 7:**
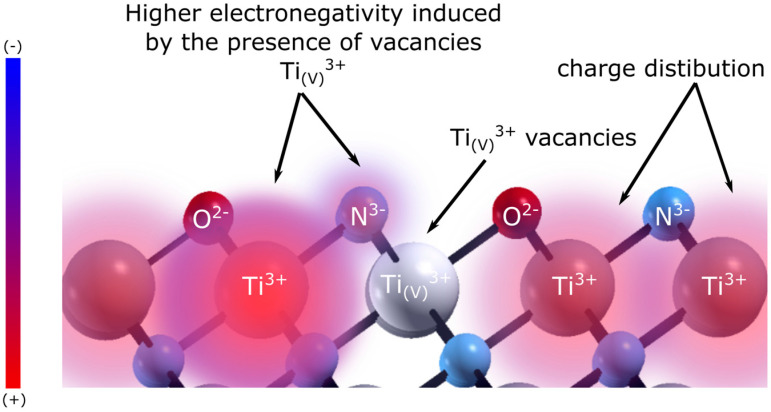
Schematic illustration of the electronic changes Ti^3+^ ions in the vicinity of the defect becoming more negative. The figure is color coded according to the change in atomic charge, with a color gradient from positive (red) to negative (blue).

Following the reasoning of Roy et al. [32] and the schematic Figure 7, the existence of these vacancies results in the Ti^3+^ ions in the vicinity of the defect becoming more negative because they donate electrons to the electronegative N^3−^ and O^2−^ ions. A similar reasoning might be applied to those nitrogen ions adjacent to the oxygen defects. As hydrogen adsorbs both on Ti and N sites, we may expect that altering the electronic density of these sites also alters the hydrogen adsorption energy on them, possibly leading to more active sites, which explains the higher performance of the TiNO layer. These assumptions are corroborated by the DFT calculations described below. 

### 4.2. DFT Calculations

DFT is an approach that allows the electronic structure and energetics of materials to be studied accurately. It provides insight into the distribution of electrons and calculations of the total energy of a given system. DFT has become a powerful tool for simulating the interaction between hydrogen molecules and solid surfaces [41]. When hydrogen molecules interact with TiN surfaces, several factors come into play. These include the binding energy between hydrogen and TiN, charge transfer, geometric configurations, and the effect of surface defects and impurities. DFT calculations of the adsorption energy of hydrogen on TiN and TiNO have presently allowed us to test the validity of the assumptions outlined above.

**Figure 8:**
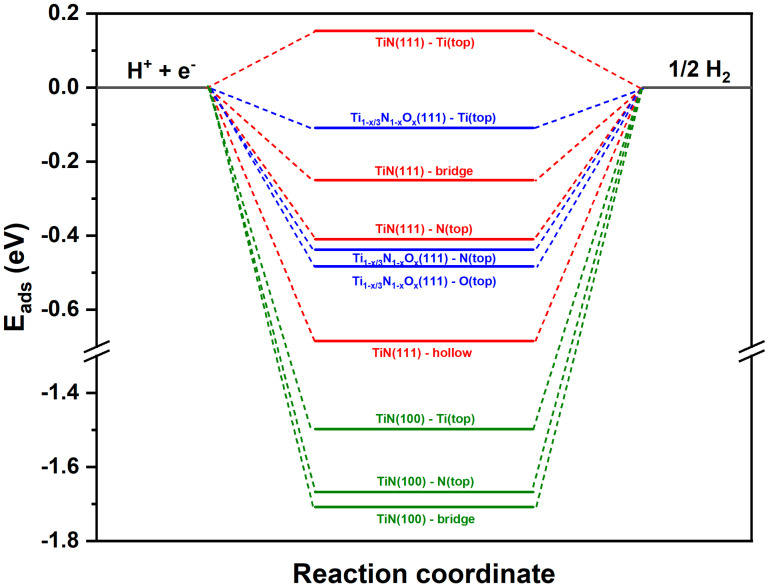
Hydrogen adsorption energy profile of the HER on various sites of TiN (100), TiN (111), and Ti_(1−x/3)_N_1−x_O_x_ (111).

Several important observations have been made regarding hydrogen adsorption on TiN surfaces. Hydrogen bonding energy represents the strength of the bond between hydrogen molecules and the surface of catalyst materials during the adsorption process. Hydrogen molecules tend to bind strongly to TiN (100) surfaces compared to TiN (111), with a difference of ~1 eV, favoring the bridge sites of TiN (100) and the hole sites of TiN (111) (see Figure 8). This difference in adsorption energy weakens the ability to release hydrogen from the metal surface, increasing hydrogen poisoning and blocking active sites. Intermediate hydrogen adsorption energies play, however, a central role in hydrogen production. An optimal intermediate hydrogen adsorption energy is important for several reasons [32]. First, it controls the efficiency of hydrogen evolution reactions, such as water splitting or feedstock hydrogenation, which are fundamental processes in green hydrogen production. Catalysts with the right adsorption energy can lower the activation barriers for these reactions, enabling faster and more cost-effective hydrogen production. Second, controlling this energy allows engineers and scientists to design catalyst materials that are selective, stable, and resistant to poisoning, improving the durability of hydrogen production systems. Surface defects, such as vacancies or impurities, can enhance or prevent hydrogen adsorption, emphasizing the importance of surface quality in real-world applications. To compare the results of the calculations with the experiments, oxygen impurities were introduced. The hydrogen adsorption energies were calculated at the different sites for Ti_(1−x/3)_N_1−x_O_x_ (111). Due to the changes caused by the oxygen defects, a reduced adsorption energy is expected. The difference between nitrogen and oxygen in terms of electronic structure results in a redistribution of charge on the surface. This redistribution enhances hydrogen adsorption on titanium, leading to an intermediate bonding of −0.1 eV, a value that, if not in the range of the −0.04 eV known for platinum [42], is nevertheless rather promisingly low to point to Ti_(1−x/3)_N_1−x_O_x_ as a very good HER catalyst. The results obtained advantageously compare to those of more sophisticated catalysts such as (Pd-O)-co-doped MoS_x_ [43], MoS_2_ nanosheets [19], a non-metallic hybrid catalyst based on graphitic C_3_N_4_, and nitrogen-doped graphene [20], while simultaneously affording superior opportunities with regards to scalability, ease of preparation, etc.

**Figure 9:**
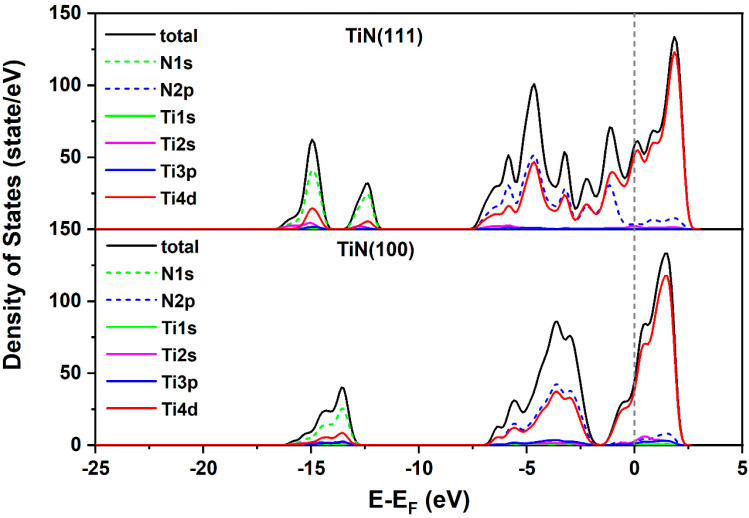
Orbital electronic density of states for TiN (111) and TiN (100). E_F_ denotes the Fermi level.

**Figure 10:**
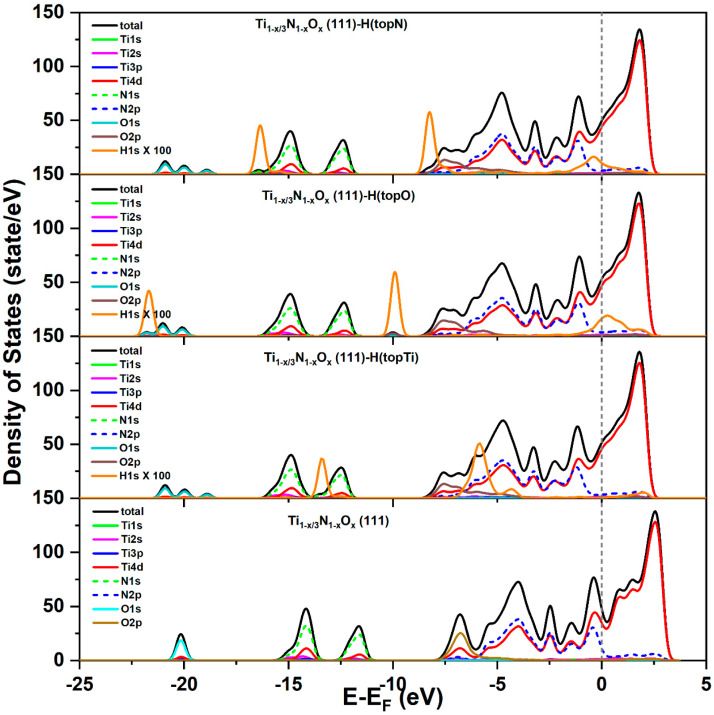
Orbital electronic density of states for Ti_(1−x/3)_N_1−x_O_x_ (111), and hydrogen atoms adsorbed on different sites Ti, N, and O. The H density of states is magnified by 100 for comparison. E_F_ denotes the Fermi level.

The density of states for TiN (111) (Figure 9) reveals a continuous state distribution spreading over a large range of 7.5 eV compared to TiN (100) (Figure 9), which shows a discontinuity around −1.8 eV, affecting the charge transfer from TiN to hydrogen by adsorption. Due to the few states between −1.8 eV and the Fermi level for TiN (100), more energy is required to share the electron from a deeper energy level with hydrogen, which is the opposite case for TiN (111). The overall effect of oxygen on TiN density is on Ti atoms, as changes in the local environment around Ti atoms due to oxygen defects affect its catalytic activity. A closer look at Ti shows that the oxygen defects cause changes in the density of states in Ti (Figure 10); Ti4d and Ti3p are very well distributed across the energy axis. The “good” hydrogen adsorption energy is reflected in the positioning of the density of states at around −5 eV and −13 eV. TiN is a well-known refractory material with a high density of electrons in the conduction band due to the presence of nitrogen. When oxygen defects are introduced, they can act as electron acceptors, leading to the formation of titanium vacancies, mentioned above, and a shift in the electronic structure of titanium. This results in the creation of localized energy states within the band. The presence of oxygen-related states leads to changes in the electronic band structure. This change can affect the electrical conductivity of the material and its ability to participate in charge transfer processes [44]. 

In summary, the presence of oxygen impurities on TiN surfaces alters the adsorption of hydrogen on titanium by changing the electronic structure of titanium. The introduction of oxygen-related electronic states leads to changes in the charge distribution and electronic structure, which, in turn, affect the interaction between hydrogen molecules and the TiN surface. This has implications for various applications where hydrogen adsorption on TiN surfaces is important, including catalysis and hydrogen storage. Thus, strategies to introduce oxygen defects at certain levels can improve the catalytic performance of TiN.

### 4.3. Pd-Top-Layer Effects

The question that now arises is as to why Pd-NPs depress the performance of the whole structure, although Pd is known to be a potent HER catalyst. As shown in Figure 5a, sputtering a thin layer of Pd on TiN results in a marked decrease in the overall performance, despite the fact that the Rct values recorded were lower than those of TiNO. Two possible explanations having the same outcome may be suggested. The first invokes the scavenging of surface oxygen defects by the Pd-atoms, leading to the formation of PdO and possibly the annihilation of Ti_(V)_^3+^. This indeed has been observed via scanning tunneling microscopy on Pd-NPs that were deposited on non-stoichiometric TiO_2_ (that contains Ti_(V)_^3+^) [44]. A second possibility invokes the formation of an ultrathin TiO_x_ layer on the Pd-NPs [45]. Whether the first or second case applies is subject to speculation until a proper analytical method is used to discriminate between them. The outcome is, however, the same, as the electronic and defect structures of TiNO will be altered in both cases, leading to changes in the electrocatalytic performance.

## 5. Conclusions

In conclusion, TiN and TiN-PdNPs were processed on Ti-substrates via physical vapor deposition. Morphological characterization shows nanostructured, highly porous surfaces with spiky nanocrystallites. XRD investigations reveal a non-stoichiometric rock salt TiN structure, eventually Pd-NPs. The surface chemistry studied by X-ray photoelectron spectroscopy unambiguously shows the existence of oxygen defects, pointing to the formation of a TiNO phase at the surface. The suitability of the TiNO and TiNO/Pd-NPs structures as electrocatalysts for the hydrogen evolution reaction was evaluated via linear sweep voltammetry, Tafel plots, and electrochemical impedance spectroscopy. TiNO shows an outstanding performance towards HER, and DFT calculations reveal that oxygen impurities in TiN surfaces have a significant influence on hydrogen adsorption energy through their effect on the electronic structure of titanium. The introduction of oxygen-related electronic states changes the charge distribution and electronic structure, affecting the interaction of hydrogen molecules with the TiNO surface. This has important implications for applications such as catalysis and hydrogen storage, where hydrogen adsorption on TiN is critical. Introducing oxygen defects at controlled levels can improve the catalytic behavior of TiN. When a thin Pd layer is sputtered on TiNO, a drastic performance decrease is observed; this is thought to arise from the scavenging of oxygen defects by Pd atoms, entailing the formation of PdO or an ultrathin TiOx layer on the Pd-NPs, possibly removing Ti^3+^ vacancies. This is further proof of the importance of TiNO surfaces for HER performance. Thus, ensuing studies will have to quantify the effects of oxygen defects in TiN on HER by controlling their concentration. This may pave the way for a high-performance catalyst that is, at the same time, cheap, robust, and easily scalable.

## Figures and Tables

**Table 1:** XPS binding energies after deconvolution of N1s and O1s and assignment to the different bonds [32,33,34,35].

Peak	Binding Energy (eV)	Assignment
N1s	397.3	TiN
	399.2	Ti-O-N
	400.2; 401.3	N-O_x_; adsorbed N_2_
	530.4	TiO_2_
O1s	531.9	Defective oxide TiO_x_
	532.7	Hydroxyl

**Table 2:** The values of R_s_ and R_ct_ calculated using a reactive system as the model.

Overvoltage (V)	R_s_ (Ohm)		R_ct_ (Ohm)	
	Ti/TiN	Ti/TiNO-PdNPs	Ti/TiN	Ti/TiNO-PdNPs
−0.4	3.36	3.45	1.121	0.52
−0.3	3.48	3.32	1.845	0.76
−0.2	3.51	3.33	5.477	1.16
−0.1	3.52	3.35	38.97	2.18

## Data Availability

Data are contained within the article.

## Supplementary Materials

The following supporting information can be downloaded at: https://www.mdpi.com/article/10.3390/nano14090770/s1, Figure S1: a photograph of a large-area thin film; Figure S2: morphology of the films after the HER experiments.
