# Supplementary material for: Oxygen Defects Containing TiN Films for the Hydrogen Evolution Reaction: A Robust Thin-Film Electrocatalyst with Outstanding Performance
Source: Nanomaterials (Basel). 2024 Apr 27;14(9):770. doi: 10.3390/nano14090770 (PMC11085547; doi:10.3390/nano14090770)
Supplement: Supplementary file 1 [file nanomaterials-14-00770-s001.zip › nanomaterials-2960400-supplementary.pdf]

Supplementary Materials

Oxygen Defects Containing TiN Films for the Hydrogen Evolution Reaction: A Robust Thin-Film Electrocatalyst with Outstanding Performance

Ayoub Laghrissi [1] and Mohammed Es-Souni [2,*]

[1] Currently at Mads Clausen Institute, University of Southern Denmark, 6400 Sønderborg, Denmark; laghris-si@mci.sdu.dk
[2] Institute of Materials and Surface Technology, Honorary Member of Kiel University of Applied Sciences, 24249 Kiel, Germany
* Correspondence: mohammed.es-souni@fh-kiel.de

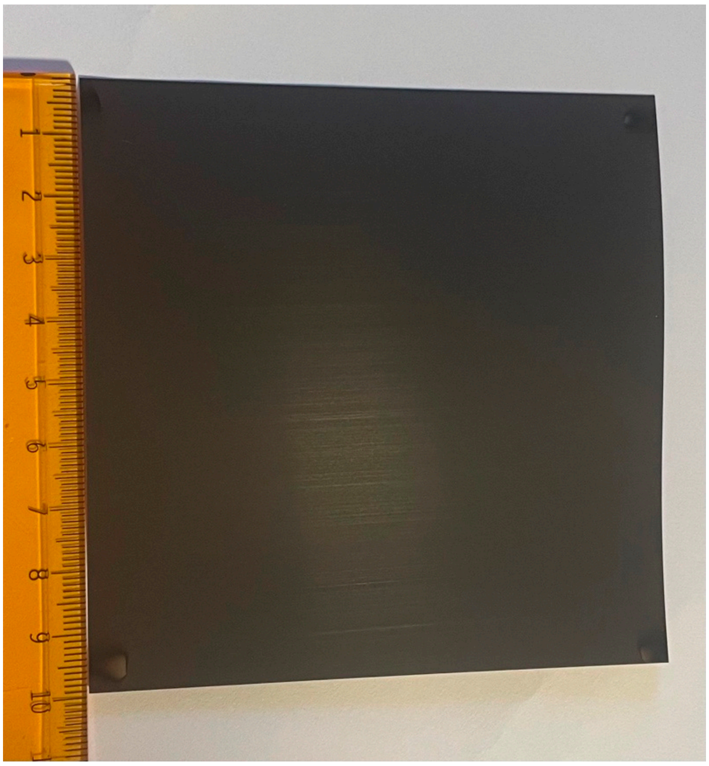

Figure S1: a photograph of a large area TiNO film deposited on a Ti sheet.

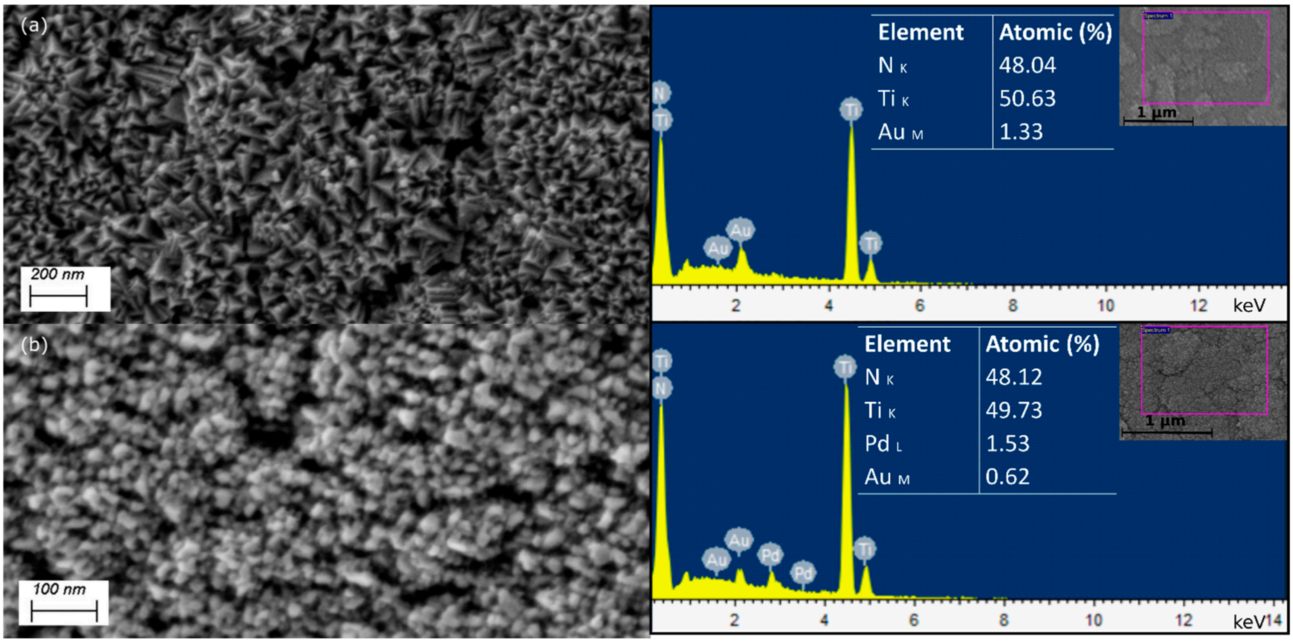

Figure S2: Top view secondary electron (SE) micrographs of TiN (**a**) and TiN-PdNPs (**b**) together with their EDS analysis after HER studies.
